# Willingness of Older Adults with Chronic Diseases to Receive a Booster Dose of Inactivated Coronavirus Disease 2019 Vaccine: A Cross-Sectional Study in Taizhou, China

**DOI:** 10.3390/vaccines10101665

**Published:** 2022-10-06

**Authors:** Xiao-Qing Lin, A-Li Li, Mei-Xian Zhang, Li Lv, Yan Chen, He-Dan Chen, Tao-Hsin Tung, Jian-Sheng Zhu

**Affiliations:** 1 Department of Infectious Diseases, Taizhou Hospital of Zhejiang Province Affiliated to Wenzhou Medical University, Linhai 317000, China; 2 Evidence-Based Medicine Center, Taizhou Hospital of Zhejiang Province Affiliated to Wenzhou Medical University, Linhai 317000, China; 3 Department of Infectious Diseases, Taizhou Hospital, Zhejiang University, Linhai 317000, China

**Keywords:** booster dose, China, chronic disease, COVID-19 vaccine, older adults, willingness

## Abstract

Vaccination is an important measure to control the spread of COVID-19 among elderly high-risk groups; however, the propensity to receive COVID-19 vaccine boosters has not been evaluated in these populations. Here, we aimed to investigate the willingness to receive a COVID-19 vaccine booster among the elderly chronic disease population in Taizhou, China. A cross-sectional, hospital-based survey was conducted in the outpatient department of a tertiary care hospital between 6 July and 11 August 2021 in Taizhou, China, and the data were uploaded to Wen-Juan-Xing, one of the largest online platforms used to collect survey data in China. The targeted population was non-oncology chronic disease patients aged 60 years and above. The minimum sample size was 229, determined by the G*Power software (v3.1.9.2, Heinrich-Heine-Universität Düsseldorf, Düsseldorf, Germany). A total of 254 patients with valid data were enrolled in this study, with a response rate of 82.5% (254/308). Chi-square tests and one-way binary regression were used to compare the proportions and the degree of influence of categorical factors. The magnitude of the effect for the comparisons was measured by Gramer’s V. A multivariate binary logistic regression model was used to correct for confounders and to identify factors. All data were analyzed using SPSS v24.0 (IBM Corporation, Armonk, NY, USA). A total of 198 respondents (77.9%) were willing to receive a COVID-19 vaccine booster dose, and 77.6% of respondents were willing to receive the primary dose. Age < 70 years (OR 2.82), stable disease control (OR 2.79), confidence in the effectiveness of the COVID-19 vaccine (OR 3.11), and vaccine recipient (OR 5.02) were significantly associated with the willingness to receive a COVID-19 vaccine booster dose. Promoting primary dose vaccination is essential for advancing booster vaccination, and it is important to focus on elderly patients’ confidence in the vaccine, in addition to strengthening health management and promoting disease stability. Follow-up studies should focus on elderly patients who belong to specific disease groups.

## 1. Introduction

Since the COVID-19 global pandemic, more than 600 million people have been diagnosed and, as of 16 September 2022, more than 6 million people have died worldwide. Vaccination is an important measure to control the spread of COVID-19 [1]. Vaccination against COVID-19 is even more critical in elderly high-risk groups [2]. Some studies have shown that a vaccination strategy that focuses on patients with chronic diseases and the elderly can minimize the number of deaths [3,4]. However, the emergence of mutant strains of COVID-19 with increasing immune escape capacity and “breakthrough infection” poses a significant challenge to outbreak control [5], especially for older adults with chronic diseases. As compared with younger people, older people with chronic illnesses aged >60 years have a higher prevalence of COVID-19 [6,7]; the severity of the infection is worse, and the mortality rate is higher [8]. The immune system of the elderly gradually declines with age, and the combination of chronic diseases can lead to more severe immune deficiencies and dysfunctions [9,10]. Therefore, prevention and control in the current outbreak is focused on encouraging elderly patients with chronic diseases to receive a timely COVID-19 vaccine booster to achieve adequate vaccine immunization and effectively prevent SARS-CoV-2 infection.

Numerous studies have focused on consolidating the protective efficacy of the COVID-19 vaccine and strengthening individual immunity, especially for people at high risk of infection [11,12]. Several studies have shown that individuals who received two doses of inactivated vaccines had an improved immune memory. A booster vaccination is essential to supplement the decreasing protective efficacy of the vaccine against the emergence of mutated strains, which, in turn, would protect the vulnerable elderly disease populations from infection and development of severe disease and would reduce the socioeconomic burden. Moreover, a booster dose of COVID-19 vaccine re-enforces individual immune responses in the elderly population and enhances the protective effect of the vaccine against a variety of COVID-19 variants, including Delta variant strains [13,14,15], providing a scientific basis for booster vaccination. In the process of promoting COVID-19 vaccine booster doses in various countries, older adults over 60 years of age and people with comorbid underlying diseases are the priority groups currently recommended for booster vaccination. China has also released a COVID-19 booster vaccination strategy for the key populations.

There are few studies on the willingness to receive the COVID-19 vaccine booster that have focused on the population of older adults with chronic diseases; studies have mostly been focused on the general adult population and healthcare workers [13,14]. Questionnaires have also been widely used as a research method in previous studies on the intention to vaccinate the elderly with the initial doses of the COVID-19 vaccine. An existing study has shown that bad health conditions can reduce the willingness of older adults to receive the initial dose of COVID-19 vaccine [15].

In this study, we aimed to investigate the willingness of a China’s elderly chronic disease population to receive a booster dose of COVID-19 vaccine, and further, to analyze the factors related to their willingness. In addition, we provided a basis for promoting COVID-19 booster vaccination in the future to improve the extent of immune protection offered by the COVID-19 vaccine.

## 2. Patients and Methods

### 2.1. Study Design and Data Collection

A cross-sectional, hospital-based survey was conducted in the outpatient department of a tertiary care hospital in Taizhou, China, and then it was uploaded to Wen-Juan-Xing, one of the largest online platforms used to collect survey data in China. We conducted a questionnaire survey in the geriatric outpatient department of Taizhou Hospital between 6 July and 11 August 2021. A total of 308 patients received the invitation. The participants voluntarily completed a self-administered questionnaire by scanning a quick response code on their mobile smartphones. The sample size was determined by using the G*Power software (v3.1.9.2, Heinrich-Heine-Universität Düsseldorf, Düsseldorf, Germany) [16,17]. We used 2-sided testing, odds ratio = 2, Pr (Y = 1| X = 1) H0 = 0.5, α err prob = 0.05, power (1-β err prob) = 0.85, R^2^ other X = 0.6. The minimum sample size was computed to be 229. The inclusion criteria for this study were patients aged 60 years and above with non-oncological chronic diseases. Respondents who did not answer completely or contained unreasonable information were excluded. A total of 254 patients with valid data were enrolled in this study, with a response rate of 82.5% (254/308). The study was approved by the Ethics Committee of Taizhou Hospital, Zhejiang Province, China (approval number K20210705). All procedures were performed according to the guidelines of the ethics committee of the authors’ institution and adhered to the tenets of the Declaration of Helsinki.

### 2.2. Structured Questionnaires and Measurement

The online self-administered questionnaire consisted of several sections. The introduction to the questionnaire described the background and purpose of the survey and indicated that the questionnaire was answered anonymously and voluntarily with informed consent. Questionnaires from previously published articles by our team were used as references [18], and the initial questionnaire for this study was formed by a meeting with experts. There was a pilot study performed. In order to ensure the quality of the official questionnaire, 20 elderly patients with chronic diseases were invited to participate in an initial test at the hospital outpatient department, and then questions that were deemed unreliable were revised based on feedback from the test population. The final version of the questionnaire for this study was confirmed through meetings with experts again.

The questionnaire consisted of several sections. The first section contained basic demographic information including age, gender, residence, education level, occupation, and perceived risk of COVID-19. We also investigated the chronic disease status of the patients. Underlying chronic disease included hypertension, diabetes disease, hyperlipidemia, hyperuricemia, chronic liver disease, chronic kidney disease, chronic pulmonary disease, cardiovascular disease, and cancer (two items: yes, no). In the final analysis, after excluding cancer patients, we recorded those with only one chronic disease as “no comorbidity” and those with two or more chronic diseases as “more than one comorbidity”. The stability of chronic diseases was measured using the question, “What is your current chronic disease status?” (two items: unstable, stable). Patients were also asked whether clinical treatment was received (two items: yes, no).

Two questions assessed the primary dose COVID-19 vaccination status: (1) Have you received the primary doses of the COVID-19 vaccine? (two items: yes, no); (2) If not yet vaccinated, are you willing to get vaccinated? (two items: yes, no). This questionnaire investigated the willingness of elderly patients with chronic diseases to receive a booster dose of the COVID-19 vaccine. In addition, their perceptions of the efficacy and safety of the COVID-19 vaccine booster were also included. The following question measured the willingness of respondents to receive a booster vaccination: “Would you be willing to receive a booster dose of COVID-19 vaccine to improve its protective effect?” The response options were strongly willing, willing, unwilling, or strongly unwilling. To facilitate the analysis, the possibilities were categorized so that the first two options were classified as “willing” and the other options were classified as “unwilling”. In addition, confidence in the effectiveness of the COVID-19 vaccine booster against COVID-19 was assessed using the following four-point Likert scale: very good, relatively good, moderate, or very poor. Confidence in safety was evaluated using the following five-point Likert scale: very safe, safe, moderate, unsafe, or very unsafe. In the final analysis, the first two options were recoded as applicable (high), while the other options were recoded as not useful (low).

### 2.3. Statistical Analysis

First, the intention to vaccinate against COVID-19 was expressed as a percentage. Second, categorical variables regarding essential demographic characteristics were expressed as counts and percentages. To assess differences between the willing and unwilling groups, chi-square tests and one-way binary regression were used to compare the proportions and the degree of influence of categorical factors. The magnitude of the effect for the comparisons was measured by Gramer’s V. A multivariate binary logistic regression model was used to correct for confounders and to identify factors that might influence the willingness to receive a booster dose of the vaccine. All logistic regression models calculated odds ratios (ORs) and corresponding 95% confidence intervals (CIs). All data were analyzed using SPSS version 24.0 (IBM Corporation, Armonk, NY, USA). Differences were considered to be statistically significant at *p* < 0.05.

### 2.4. Literature Search Strategy 

A search was performed in The Cochrane Library, PubMed, and EMBASE databases for relevant studies from inception to 16 September 2022. Searches included a mix of MeSH and free-text terms related to the key concepts of willing, older, adults, vaccine, and COVID-19, with no language restrictions. After filtering out the irrelevant literature and removing the duplicate literature by scanning the titles, abstracts, and full text, 9 related papers were finally obtained. We extracted the following data from the included studies using a data-extraction form: first author, country, study period, study design, age, sample size, amount of willing to vaccinate the primary dose of COVID-19 vaccine, and main factors associated with more willing to be vaccinated.

## 3. Results

### 3.1. Demographic Characteristics of the Study Population

Table 1 summarizes the characteristics of the older patients with chronic diseases surveyed, and 254 questionnaires were analyzed in the present study. Among the participants, 66.1% of the participants were aged 60–69 years, 33.9% were aged >70 years, and 56.3% were male. Among the older adults, 47.6% had a combination of multiple chronic diseases, and the majority (87.4%) had stable disease control. There were 89 (35.0%) respondents who had been vaccinated with the primary dose of COVID-19 vaccine. Moreover, 108 of those who were not vaccinated were willing to be vaccinated in the future. Therefore, the total proportion of participants willing to be vaccinated was 77.6%.

### 3.2. The Willingness of Older Adults with Chronic Disease to Receive a Booster Dose of COVID-19 Vaccine

**Figure 1:**
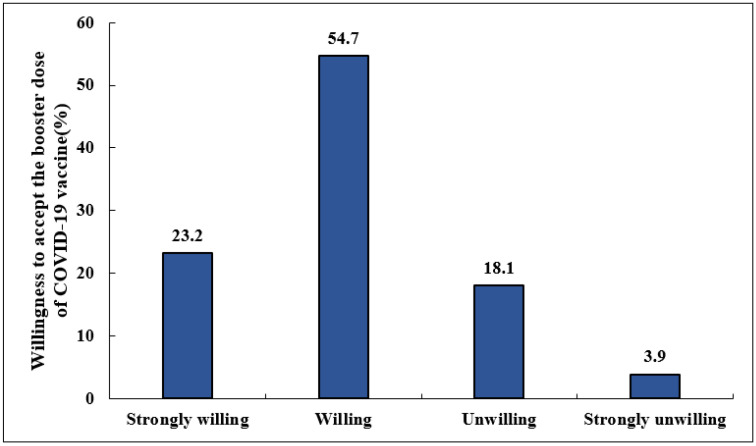
The willingness of older adults with chronic disease to receive a booster dose of COVID-19 vaccine, *n* = 254.

**Figure 2:**
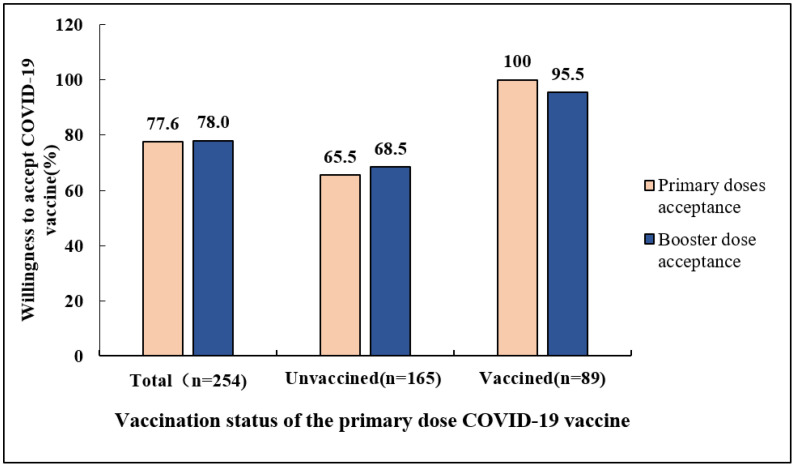
Willingness to accept COVID-19 vaccine in vaccinated and unvaccinated participants, *n* = 254.

As shown in Figure 1, 198 (77.9%) respondents were willing to receive a booster dose of COVID-19, among which 139 (54.7%) were willing, and 59 (23.2%) had a strong desire to receive a vaccine booster. In contrast, 56 (22.1%) respondents indicated that they were unwilling or strongly unwilling to accept a booster dose. As shown in Figure 2, among the older patients with chronic diseases surveyed, the proportion of them willing to receive a booster dose of COVID-19 was not significantly different from that of the primary dose (78.0% vs. 77.6%, *p* > 0.05). Interestingly, 68.5% of participants who did not receive the primary dose of the COVID-19 vaccine reported a willingness to accept a booster shot. However, this was significantly lower than in those who had already received the vaccine (68.5% vs. 95.5%, *p* < 0.001).

### 3.3. Factors Associated with Willingness to Accept a Booster Dose of COVID-19 Vaccine

Table 2 summarizes the potential factors influencing the willingness of elderly patients with chronic diseases to receive a COVID-19 booster vaccine. There were no significant differences between those willing and unwilling to accept vaccine boosters in terms of gender, place of residence, education level, or occupation (*p* > 0.05). Respondents with multiple comorbid chronic diseases and unstable disease control had significantly lower willingness to receive COVID-19 vaccine boosters. Those with low confidence in the safety and efficacy of the COVID-19 vaccine and those who had not received the vaccine were more reluctant to receive a booster (all *p* < 0.05).

We further calculated the degree of association between these factors and the willingness to receive booster vaccination using a binary logistic regression model. As shown in Table 3, age < 70 years (OR 2.82, 95% CI 1.37–5.81), stable disease control (OR 2.79, 95% CI 1.11–7.00), confidence in the effectiveness of the COVID-19 vaccine (OR 3.11, 95% CI 1.34–7.22), and vaccine recipient (OR 5.02, 95% CI 1.67–15.14) were significantly associated with willingness to receive a booster dose of COVID-19 vaccine. After stratification by vaccination status, the correlates of willingness to accept a booster dose in the vaccinated subsample were consistent with the entire sample.

### 3.4. The Estimates of Older Adults’ Willingness to Receive the Primary Doses of the COVID-19 Vaccine

We summarized the different estimates of older adults’ willingness to receive the primary dose of the COVID-19 vaccine in Table 4 [15,19,20,21,22,23,24,25,26]. Willingness estimates ranged from 43.9% to 92.7%. The results of our study showed that 77.6% of older adults with chronic diseases were willing to receive the primary dose of COVID-19 vaccine, which was in line with previous findings.

## 4. Discussion

### 4.1. Clinical Implications

As elderly individuals form one of the high-risk groups for COVID-19, COVID-19 vaccination in the elderly is a hot topic of current research. Studies have shown the immunization effects of different types of COVID-19 vaccines in elderly people [27,28,29]. A clinical study of a ChAdOx1 nCoV-19 vaccine booster showed that the immunogenicity of the booster in older adults was similar to that of younger adults, enabling a robust humoral and cellular immune response in the older population [30].

A comparison of the willingness of older adults with chronic diseases to receive the booster dose and its associated factors with the existing studies (Table 4) of the primary doses for the general elderly revealed that the willingness of older adults in different countries ranged from 43.9% to 92.7%, and the results of our study showed that age groups > 60 years with chronic diseases also had a high rate of willingness to receive the COVID-19 vaccine booster (77.9%), similar to their willingness to receive the primary dose (77.6%). In an Italian study, the elderly showed good knowledge about COVID-19 and its preventive aspects [31]. The perceptions of the COVID-19 vaccine, particularly safety and efficacy, were positively associated factors in the willingness to receive the initial dose in the general elderly population in various countries. Our study found that willingness to receive a booster dose in the elderly chronic disease patient population was similarly influenced by perceptions of COVID-19 vaccine safety and efficacy.

There are also different findings in this survey. First, The results of this study showed that 95.5% of those who completed the initial dose of the COVID-19 vaccine were willing to receive the booster dose. Therefore, the vaccination strategy for the booster dose should be focused on increasing the acceptance of the initial dose of the vaccine and encouraging individuals who have not yet received the initial dose to get vaccinated as soon as possible, thereby, increasing the booster dose administration and maintaining the continuity of herd immunity. Second, the impact of older age and clinical stability of disease control on the willingness of older adults with chronic conditions to receive COVID-19 vaccine boosters is not negligible.

Our findings showed that older adults aged >70 years were significantly less willing to receive a booster dose than older adults aged <70 years and well below the requirements for group immunization [32], which may be related to the lack of awareness and confidence in the COVID-19 vaccine among older adults. This suggests that knowledge of the vaccine affects older adults’ willingness to vaccinate. However, the level of understanding among older adults decreases with age. Additionally, older adults may have less access to COVID-19 vaccine-related knowledge due to decreased self-care and social participation, thereby, reducing the level of knowledge about the effectiveness of the booster [33]. Perceptions of and attitudes toward COVID-19 risk vary by age group, with older adults having more negative attitudes toward vaccination. However, high infection and mortality rates of COVID-19 occur more frequently in older adults [34]. Therefore, during COVID-19, society, communities, and families need to pay special attention to the older age groups to enhance their education about the disease and to promote their awareness of the vaccine to improve their acceptance of COVID-19 vaccine boosters [35]. Healthcare professionals have a close and trusting relationship with older patients with chronic diseases and are a reliable source to help patients to understand vaccine information [36]. Therefore, enhancing the involvement of healthcare professionals in COVID-19 prevention efforts will help to increase confidence in booster doses among patients with chronic diseases.

Most elderly patients have chronic diseases and multiple comorbidities [37]. Therefore, the degree of disease control has important implications for the willingness to receive COVID-19 vaccine booster shots. Our study showed that patients with multiple comorbidities, especially those strongly associated with COVID-19 complications, were significantly less willing to accept COVID-19 vaccine booster shots. According to the existing recommendations for COVID-19 vaccination in patients with chronic diseases, medical stability is the basis for vaccination [4]. Patients with chronic disease are more likely to follow the rules, guidelines, and recommendations during the COVID-19 pandemic [38]. A patient’s primary condition can impact vaccine action, causing the effect of the vaccine to be reduced by a partially protective immune response [39]. Successful and effective health management strategies can increase the motivation and initiative of older adults to address their health problems [40], thus, potentially increasing their willingness to receive booster shots. Therefore, strengthening the scientific management of chronic diseases in elderly patients to help them maintain and improve their health is an important strategy to promote COVID-19 booster vaccination.

There are some practical recommendations based on this study. First, hospitals can provide individualized health education according to the different conditions of patients. The focus should be on health education for elderly people with stable chronic disease conditions and urging them to get booster vaccinations as soon as possible. In addition, for patients whose condition is in an unstable stage, the main focus is on treating the disease. Secondly, the government can develop incentives and establish cooperation with hospitals to precisely intervene in people who are suitable for COVID-19 booster vaccination.

### 4.2. Methodological Considerations

There are some limitations to our study. First, we recruited chronic disease patients from hospitals in a single geographic area in Taizhou, China. The study population was also selected on a convenient and voluntary basis. Therefore, inevitable selection bias existed in this study. Second, the survey was conducted using a non-standardized questionnaire. Since our questionnaire used a non-scale format, it was difficult to test the reliability, intelligibility, and validation indexes. Third, patients’ stability of chronic diseases was assessed by their self-assessment and it was measured by a single-choice question. Moreover, the evaluation criteria for disease stability varied among chronic diseases. Therefore, there may have been some measurement bias in the results of stability of chronic diseases. Fourth, our study did not analyze the elderly group with specific diseases, which is the direction of our follow-up study.

## 5. Conclusions

Our study found that 77.9% of older adults with chronic diseases were willing to receive a booster dose of the COVID-19 vaccine, which was similar to their willingness to accept the primary doses. Furthermore, 95.5% of those who completed the primary doses were willing to receive a booster dose. Therefore, promoting primary doses of vaccine is essential for advancing booster vaccination. The willingness to receive the booster dose was significantly lower in elderly patients > 70 years with chronic diseases and unstable conditions. This emphasizes the importance of educating the elderly with chronic diseases, with particular attention to their confidence in vaccines. Further, the health management of chronic diseases in the elderly should be strengthened to promote disease stabilization. These are essential strategies for promoting booster doses of vaccination in this particular population.

## Figures and Tables

**Table 1:** Demographic characteristics of the study population, *n* = 254.

Independent Variables	Categories	n (%)/Mean ± SD
Total		254 (100%)
Age	67.9 ± 6.8	
Sex	Male	143 (56.3%)
	Female	111 (43.7%)
Residence	Rural	164 (64.6%)
	Urban	90 (35.4%)
Education level	Junior high school and below	199 (78.3%)
	High school and above	55 (21.7%)
Occupation	Farmer	157 (61.8%)
	Workmen	27 (10.6%)
	Others	70 (27.6%)
Risk perception of COVID-19	High	72 (28.3%)
	Low	182 (71.7%)
Have you vaccinated primary dose of vaccine against COVID-19	Yes	89 (35.0%)
	No	165 (65.0%)
If not vaccinated, willing to be vaccinated against COVID-19	Yes	108 (42.5%)
	No	57 (22.4%)
Underlying chronic diseases	Hypertension	184 (72.4%)
	Diabetes	106 (41.7%)
	Hyperlipidemia	56 (22.0%)
	Hyperuricemia	63 (24.8%)
	Liver	60 (23.6%)
	Kidney	119 (46.9%)
	Pulmonary	25 (9.8%)
	Cardiovascular	59 (23.2%)
Comorbidity	No comorbidity	69 (27.2%)
	One comorbidity	64 (25.2%)
	More than one comorbidity	121 (47.6%)
Stability of chronic diseases	Unstable	32 (12.6%)
	Stable	222 (87.4%)
Are you in clinical treatment?	Yes	217 (85.4%)
	No	37 (14.6%)

**Table 2:** Cardinal analysis of factors influencing the acceptability of COVID-19 vaccine booster vaccination among older adults with chronic diseases, *n* = 254.

Independent Variables	Categories	Willing to be Vaccinated		Unwilling to be Vaccinated		χ2	*p*	Gramer’s V
		198	78.0%	56	22.0%			
Age groups	60–69 years	141	83.9%	27	16.1%	10.310	0.001	0.201
	≥70 years	57	66.3%	29	33.7%			
Sex	Male	109	76.2%	34	23.8%	0.569	0.451	0.047
	Female	89	80.2%	22	19.8%			
Residence	Rural	132	80.5%	32	19.5%	1.731	0.188	0.083
	Urban	66	73.3%	24	26.7%			
Education level	Junior high school and below	158	79.4%	41	20.6%	1.115	0.291	0.066
	High school and above	40	72.7%	15	27.3%			
Occupation	Farmer	127	80.9%	30	19.1%	3.561	0.169	
	Workmen	22	81.5%	5	18.5%			0.118
	Others	49	70.0%	21	30.0%			
Risk perception of COVID-19	High	61	84.7%	11	15.3%	2.679	0.102	0.103
	Low	137	75.3%	45	24.7%			
Comorbidity	No comorbidity	63	91.3%	6	8.7%	9.826	0.002	0.197
	More than one comorbidity	135	73.0%	50	27.0%			
Stability of chronic diseases	Unstable	18	56.3%	14	43.8%	10.034	0.002	0.199
	Stable	180	81.1%	42	18.9%			
Are you in clinical treatment?	Yes	166	76.5%	51	23.5%	1.835	0.176	0.085
	No	32	86.5%	5	13.5%			
Confidence in the effectiveness of the COVID-19 vaccine	High	162	87.1%	24	12.9%	33.801	<0.001	0.365
	Low	36	52.9%	32	47.1%			
Confidence in the safety of the COVID-19 vaccine	High	169	84.5%	31	15.5%	23.464	<0.001	0.304
	Low	29	53.7%	25	46.3%			
Have you already accepted primary doses of the COVID-19 vaccine?	No	113	68.5%	52	31.5%	23.014	<0.001	0.311
	Yes	85	95.5%	4	4.5%			

**Table 3:** Logistic regression of factors associated with willingness to accept a booster dose of COVID-19 vaccine, *n* = 254.

Independent Variables		Pc	OR (95% CI)	Padj	OR (95% CI)
Age	60–69	0.001	2.66 (1.45–4.88)	0.005	2.82 (1.37–5.81)
	≥70 years	1		1	
Stability of chronic diseases	Stable vs. Unstable	0.002	3.33 (1.54–7.24)	0.029	2.79 (1.11–7.00)
Comorbidity	No comorbidity	0.002	3.89 (1.58–9.55)	0.124	2.22 (0.80–6.11)
	More than one comorbidity	1		1	
Confidence in the effectiveness of the COVID-19 vaccine	High vs. Low	0.000	6.00 (3.16–11.39)	0.008	3.11 (1.34–7.22)
Confidence in the safety of the COVID-19 vaccine	High vs. Low	0.000	4.70 (2.43–9.07)	0.175	1.84 (0.76–4.41)
Have you already accepted primary doses of the COVID-19 vaccine?	Yes vs. No	0.000	9.78 (3.40–28.09)	0.004	5.02 (1.67–15.14)

Pc: unadjusted *p*-value in binary logistic regression models without covariates; cOR (95% CI), crude odds ratio and 95% confidence interval in the binary logistic regression models without covariates; Padj, adjusted *p*-value in binary logistic regression model including all the variables; aOR (95% CI), adjusted odds ratio and 95% confidence interval in binary logistic regression model including all the variables.

**Table 4:** The estimates of older adults’ willingness to receive the primary dose of the COVID-19 vaccine.

Author	Country	Study Period	Study Design	Age (Years)	Sample Size	Willing to Vaccinate (%)	Main Factors Associated with More Willing to be Vaccinated	Reference
Wu et al.	China	1 May 2021–30 June 2021	cross-sectional	≥60	1067	90.9%	1. Perceived the safety and necessity of the COVID-19 vaccine.	[15]
Nikolovski et al.	USA	1 January 2020–31 January 2020	clinical study	≥65	7420	91.3%	1. Perceived the safety and efficacy of COVID-19 vaccines; 2. Positive results from the first COVID-19 vaccine outcome study.	[19]
Basta et al.	Canada	29 September 2020–29 December 2020	longitudinal Study	≥50	23819	84.1%	1. Perceived about the safety and efficacy of COVID-19 vaccines.	[20]
Syan et al.	Canada	15 January 2021–15 February 2021	cross-sectional	≥50	314	83.1%	1. Perceived the safety of the COVID-19 vaccine.	[21]
Galle et al.	Italy	1 June 2021–31 August 2021	cross-sectional	≥65	1041	92.7%	1. Having social/mass media as a main source of information; 2. Higher educational level.	[22]
Contoli et al.	Italy	August 2020–31 January 2021	cross-sectional	≥65	1876	54.9%	1. Received vaccination against influenza; 2. High risk perception of having had a death from COVID-19.	[23]
Macinko et al.	Brazilian	1 May 2020–30 November 2020	cross-sectional	≥50	6584	71%	1. High risk perception of COVID-19; 2. Perceived the safety and benefits of COVID-19 vaccines.	[24]
Al-Hanawi et al.	Saudi Arabia	8 December 2020–14 December 2020	cross-sectional	≥50	488	43.9%	1. Perceived the safety of COVID-19 vaccines; 2. Male; 3. Higher educational level; 4. High risk perception of COVID-19.	[25]
Malesza et al.	German	4 January 2021–17 January 2021	cross-sectional	≥75	1037	78.2%	1. High risk perception of COVID-19; 2. Perceived the safety, efficacy, and benefits of COVID-19 vaccines.	[26]

## Data Availability

The data presented in this study are available on request from the corresponding author. The data are not publicly available due to privacy.

## References

[1] Buttenheim A.M. (2020). SARS-CoV-2 Vaccine Acceptance: We May Need to Choose Our Battles. Ann. Intern. Med..

[2] Dhama K., Patel S.K., Natesan S., Vora K.S., Yatoo M.I., Tiwari R., Saxena S.K., Singh K.P., Singh R., Malik Y.S. (2020). COVID-19 in the elderly people and advances in vaccination approaches. Hum. Vaccines. Immunother..

[3] Tran T.N., Wikle N.B., Albert E., Inam H., Strong E., Brinda K., Leighow S.M., Yang F., Hossain S., Pritchard J.R. (2021). Optimal SARS-CoV-2 vaccine allocation using real-time attack-rate estimates in Rhode Island and Massachusetts. BMC Med..

[4] Afshar Z.M., Babazadeh A., Janbakhsh A., Mansouri F., Sio T.T., Sullman M.J.M., Carson-Chahhoud K., Hosseinzadeh R., Barary M., Ebrahimpour S. (2021). Coronavirus disease 2019 (Covid-19) vaccination recommendations in special populations and patients with existing comorbidities. Rev. Med. Virol..

[5] Bian L., Gao Q., Gao F., Wang Q., He Q., Wu X., Mao Q., Xu M., Liang Z. (2021). Impact of the Delta variant on vaccine efficacy and response strategies. Expert Rev. Vaccines.

[6] Liu K., Chen Y., Lin R., Han K. (2020). Clinical features of COVID-19 in elderly patients: A comparison with young and middle-aged patients. J. Infect..

[7] Parohan M., Yaghoubi S., Seraji A., Javanbakht M.H., Sarraf P., Djalali M. (2020). Risk factors for mortality in patients with Coronavirus disease 2019 (COVID-19) infection: A systematic review and meta-analysis of observational studies. Aging Male.

[8] Liu Y., Mao B., Liang S., Yang J.-W., Lu H.-W., Chai Y.-H., Wang L., Zhang L., Li Q.-H., Zhao L. (2020). Association between age and clinical characteristics and outcomes of COVID-19. Eur. Respir. J..

[9] Wagner A., Weinberger B. (2020). Vaccines to Prevent Infectious Diseases in the Older Population: Immunological Challenges and Future Perspectives. Front. Immunol..

[10] Nath K., Viswanathan S., Upham J., Davies J., Towers M., Looke D., Pritchard A., Burel J. (2014). Clinical factors associated with the humoral immune response to influenza vaccination in chronic obstructive pulmonary disease. Int. J. Chronic Obstr. Pulm. Dis..

[11] Fowlkes A., Gaglani M., Groover K., Thiese M.S., Tyner M., Ellingson K., HEROES-RECOVER Cohorts (2021). Effectiveness of COVID-19 Vaccines in Preventing SARS-CoV-2 Infection Among Frontline Workers Before and During B.1.617.2 (Delta) Variant Predominance—Eight U.S. Locations, December 2020–August 2021. Morb. Mortal. Wkly. Rep..

[12] Milne G., Hames T., Scotton C., Gent N., Johnsen A., Anderson R.M., Ward T. (2021). Does infection with or vaccination against SARS-CoV-2 lead to lasting immunity?. Lancet. Respir. Med..

[13] Pan S.-J., Yang Y.-P., Zhang M.-X., Tung T.-H. (2022). Willingness to pay for booster dose of COVID-19 vaccine among healthcare workers in Taizhou, China. Hum. Vaccines Immunother..

[14] Tung T.-H., Lin X.-Q., Chen Y., Zhang M.-X., Zhu J.-S. (2022). Willingness to receive a booster dose of inactivated coronavirus disease 2019 vaccine in Taizhou, China. Expert Rev. Vaccines.

[15] Wu L., Wang X., Li R., Huang Z., Guo X., Liu J., Yan H., Sun X. (2022). Willingness to Receive a COVID-19 Vaccine and Associated Factors among Older Adults: A Cross-Sectional Survey in Shanghai, China. Vaccines.

[16] Kang H. (2021). Sample size determination and power analysis using the G*Power software. J. Educ. Eval. Health Prof..

[17] Cohen J. (1992). Statistical Power Analysis. SAGE J..

[18] Zhang M.X., Lin X.Q., Chen Y., Tung T.H., Zhu J.S. (2021). Determinants of parental hesitancy to vaccinate their children against COVID-19 in China. Expert Rev. Vaccines.

[19] Nikolovski J., Koldijk M., Weverling G.J., Spertus J., Turakhia M., Saxon L., Gibson M., Whang J., Sarich T., Zambon R. (2021). Factors indicating intention to vaccinate with a COVID-19 vaccine among older U.S. adults. PLoS ONE.

[20] Basta N.E., Sohel N., Sulis G., Wolfson C., Maimon G., Griffith L.E., Kirkland S., McMillan J.M., Thompson M., Raina P. (2022). Factors Associated with Willingness to Receive a COVID-19 Vaccine Among 23,819 Adults Aged 50 Years or Older: An Analysis of the Canadian Longitudinal Study on Aging. Am. J. Epidemiol..

[21] Syan S.K., Gohari M.R., Levitt E.E., Belisario K., Gillard J., DeJesus J., MacKillop J. (2021). COVID-19 Vaccine Perceptions and Differences by Sex, Age, and Education in 1,367 Community Adults in Ontario. Front. Public Health.

[22] Gallè F., Sabella E.A., Roma P., Da Molin G., Diella G., Montagna M.T., Ferracuti S., Liguori G., Orsi G.B., Napoli C. (2021). Acceptance of COVID-19 Vaccination in the Elderly: A Cross-Sectional Study in Southern Italy. Vaccines.

[23] Contoli B., Possenti V., Minardi V., Binkin N.J., Ramigni M., Carrozzi G., Masocco M. (2021). What Is the Willingness to Receive Vaccination Against COVID-19 Among the Elderly in Italy? Data From the PASSI d’Argento Surveillance System. Front. Public Health.

[24] Macinko J., Seixas B.V., Mambrini J.V.D.M., Lima-Costa M.F. (2021). Which older Brazilians will accept a COVID-19 vaccine? Cross-sectional evidence from the Brazilian Longitudinal Study of Aging (ELSI-Brazil). BMJ Open.

[25] Al-Hanawi M.K., Alshareef N., El-Sokkary R.H. (2021). Willingness to Receive COVID-19 Vaccination among Older Adults in Saudi Arabia: A Community-Based Survey. Vaccines.

[26] Malesza M., Wittmann E. (2021). Acceptance and Intake of COVID-19 Vaccines among Older Germans. J. Clin. Med..

[27] Li J., Hui A., Zhang X., Yang Y., Tang R., Ye H., Ji R., Lin M., Zhu Z., Türeci Ö. (2021). Safety and immunogenicity of the SARS-CoV-2 BNT162b1 mRNA vaccine in younger and older Chinese adults: A randomized, placebo-controlled, double-blind phase 1 study. Nat. Med..

[28] Anderson E.J., Rouphael N.G., Widge A.T., Jackson L.A., Roberts P.C., Makhene M., Chappell J.D., Denison M.R., Stevens L.J., Pruijssers A.J. (2020). Safety and Immunogenicity of SARS-CoV-2 mRNA-1273 Vaccine in Older Adults. N. Engl. J. Med..

[29] Soiza R.L., Scicluna C., Thomson E.C. (2021). Efficacy and safety of COVID-19 vaccines in older people. Age Ageing.

[30] Ramasamy M.N., Minassian A.M., Ewer K.J., Flaxman A.L., Folegatti P.M., Owens D.R., Voysey M., Aley P.K., Angus B., Babbage G. (2021). Safety and immunogenicity of ChAdOx1 nCoV-19 vaccine administered in a prime-boost regimen in young and old adults (COV002): A single-blind, randomised, controlled, phase 2/3 trial. Lancet.

[31] Gallè F., Sabella E.A., Roma P., Ferracuti S., Da Molin G., Diella G., Montagna M.T., Orsi G.B., Liguori G., Napoli C. (2021). Knowledge and Lifestyle Behaviors Related to COVID-19 Pandemic in People over 65 Years Old from Southern Italy. Int. J. Environ. Res. Public Health.

[32] Randolph H.E., Barreiro L.B. (2020). Herd Immunity: Understanding COVID-19. Immunity.

[33] Sun Z., Yang B., Zhang R., Cheng X. (2020). Influencing Factors of Understanding COVID-19 Risks and Coping Behaviors among the Elderly Population. Int. J. Environ. Res. Public Health.

[34] Bonanad C., García-Blas S., Tarazona-Santabalbina F., Sanchis J., Bertomeu-González V., Fácila L., Ariza A., Núñez J., Cordero A. (2020). The Effect of Age on Mortality in Patients With COVID-19: A Meta-Analysis With 611,583 Subjects. J. Am. Med. Dir. Assoc..

[35] Dhama K., Patel S.K., Kumar R., Rana J., Yatoo M.I., Kumar A., Tiwari R., Dhama J., Natesan S., Singh R. (2020). Geriatric Population During the COVID-19 Pandemic: Problems, Considerations, Exigencies, and Beyond. Front. Public Health.

[36] Napolitano F., Polla G.D., Capano M.S., Augimeri M., Angelillo I.F. (2020). Vaccinations and Chronic Diseases: Knowledge, Attitudes, and Self-Reported Adherence among Patients in Italy. Vaccines.

[37] Fabbri E., Zoli M., Gonzalez-Freire M., Salive M.E., Studenski S.A., Ferrucci L. (2015). Aging and Multimorbidity: New Tasks, Priorities, and Frontiers for Integrated Gerontological and Clinical Research. J. Am. Med. Dir. Assoc..

[38] Lazarus J.V., Ratzan S.C., Palayew A., Gostin L.O., Larson H.J., Rabin K., Kimball S., El-Mohandes A. (2021). A global survey of potential acceptance of a COVID-19 vaccine. Nat. Med..

[39] D’Amelio R., Asero R., Cassatella M.A., Laganà B., Lunardi C., Migliorini P., Nisini R., Parronchi P., Quinti I., Racanelli V. (2021). Anti-COVID-19 Vaccination in Patients with Autoimmune-Autoinflammatory Disorders and Primary/Secondary Immunodeficiencies: The Position of the Task Force on Behalf of the Italian Immunological Societies. Biomedicines.

[40] Lorig K.R., Holman H.R. (2003). Self-management education: History, definition, outcomes, and mechanisms. Ann. Behav. Med..

